# A novel comprehensive immune-related gene signature as a promising survival predictor for the patients with head and neck squamous cell carcinoma

**DOI:** 10.18632/aging.202842

**Published:** 2021-04-17

**Authors:** Ruihua Fang, Muhammad Iqbal, Lin Chen, Jing Liao, Jierong Luo, Fanqin Wei, Weiping Wen, Wei Sun

**Affiliations:** 1 Department of Otorhinolaryngology Head and Neck Surgery, The First Affiliated Hospital, Sun Yat-Sen University, Guangzhou 510080, Guangdong, P.R. China; 2 Institute of Otorhinolaryngology Head and Neck Surgery, Sun Yat-Sen University, Guangzhou 510080, Guangdong, P.R. China; 3 Guangzhou Key Laboratory of Otorhinolaryngology, Guangzhou 510080, Guangdong, P.R. China; 4 Guangdong Institute of Gastroenterology, The Sixth Affiliated Hospital, Sun Yat-Sen University, Guangzhou 510655, Guangdong, P.R. China; 5 Department of Anesthesia, Guangzhou First People's Hospital, The Second Affiliated Hospital of South China University of Technology, Guangzhou 510080, Guangdong, P.R. China

**Keywords:** head and neck squamous cell carcinoma, immune-related signature, prognosis, immune checkpoint molecules, bioinformatics

## Abstract

Head and neck squamous cell carcinoma (HNSCC), the most frequent subtype of head and neck cancer, continues to have a poor prognosis with no improvement. The TNM stage is not satisfactory for individualized prognostic assessment and it does not predict response to therapy. In the present study, we downloaded the gene expression profiles from TCGA database to establish a training set and GEO database for a validation set. In the training set, we developed an 10 immune-related genes signature which had superior predictive value compared with TNM stage. A nomogram including clinical characteristics was also constructed for accurate prediction. Furthermore, it was determined that our prognostic signature might act as an independent factor for predicting the survival of HNSCC patients. As for the immune microenvironment, our results showed higher immune checkpoint expression (CLTA-4 and PD-1) in low-risk group which might reflect a positive immunotherapy response. Thus, our signature not only provided a promising biomarker for survival prediction, but might be evaluated as an indicator for personalized immunotherapy in patients with HNSCC.

## INTRODUCTION

Head and neck squamous cell carcinoma (HNSCC) is the sixth most common malignant tumor. Worldwide, approximately 830,000 patients suffer from head and neck cancer and about 430,000 people die from this disease annually [1]. Approximately 75% of these cases are attributable to tobacco smoking and alcohol abuse, which are the two major risk factors for head and neck cancer [2]. Human papillomavirus (HPV) infection is also a significant etiological factor for HNSCC [3]. However, despite advances in surgery, radiotherapy and chemotherapy for the treatment of HNSCC, the 5-year overall survival rate is only 40%-50% [4]. In addition, the survival rate of advanced cancer patients is only 34.9%, primarily due to metastases and recurrence [5]. Accurately assessing the prognosis for individual patients and performing personalized treatment is of vital importance. Traditionally, American Joint Committee on Cancer (AJCC) staging system-TNM stage is the most important prognostic indicator for predicting postoperative outcome of HNSCC [6]. Nevertheless, the limitation to TNM staging in evaluating patient prognosis is gradually emerging. For example, patients with the same TNM stage and treatment regimen have different clinical outcomes and it does not predict the effectiveness of patient's treatment [7]. Therefore, it is necessary to identify a novel biomarker that can accurately predict patient prognosis and to stratify high-risk HNSCC patients for more intensive treatment regimens.

An increasing body of evidence suggests that the immune system plays a vital role in patient outcome and tumor molecular profiles may be useful for predicting clinical outcome, as well as identifying therapeutic targets [8, 9]. It is also revealed that a prognostic signature containing several to dozens of genes have laid a foundation for predicting the survival of HNSCC [10–12]. In recent years, cytotoxic T-lymphocyte-associated protein 4 (CTLA-4) and programmed cell death protein-1 (PD-1)/programmed cell death-ligand 1 (PD-L1) were found to be important immune checkpoint components, that enable tumors to escape from immune surveillance [13, 14]. With the emergence of checkpoint immunosuppressive therapy, the treatment regimens have completely changed for many advanced malignant tumors including HNSCC. However, the checkpoint blockade immune therapeutics does not respond to all patients and the observed objective response rates are in the range of only 16% to 25% [15, 16]. Therefore, an immune-related prognostic signature which can not only predicts survival, but predicts the immunotherapy response for different groups of patients is urgently need.

In this study, an immune-related prognostic signature was constructed with The Cancer Genome Atlas (TCGA) dataset and further validated for its prognostic value using the GSE41613 dataset. Moreover, the relationship between our signature, infiltrating immune cells, and immune checkpoints was determined. Finally, the mutation characteristics and Gene Set Enrichment Analysis (GSEA) of our gene signature were established. Our goal is to provide a novel molecular biomarker that more effectively predicts prognosis and is strongly associated with the immune microenvironment in HNSCC patients.

## RESULTS

### Differentially expressed genes and functional enrichment analysis

**Figure 1:**
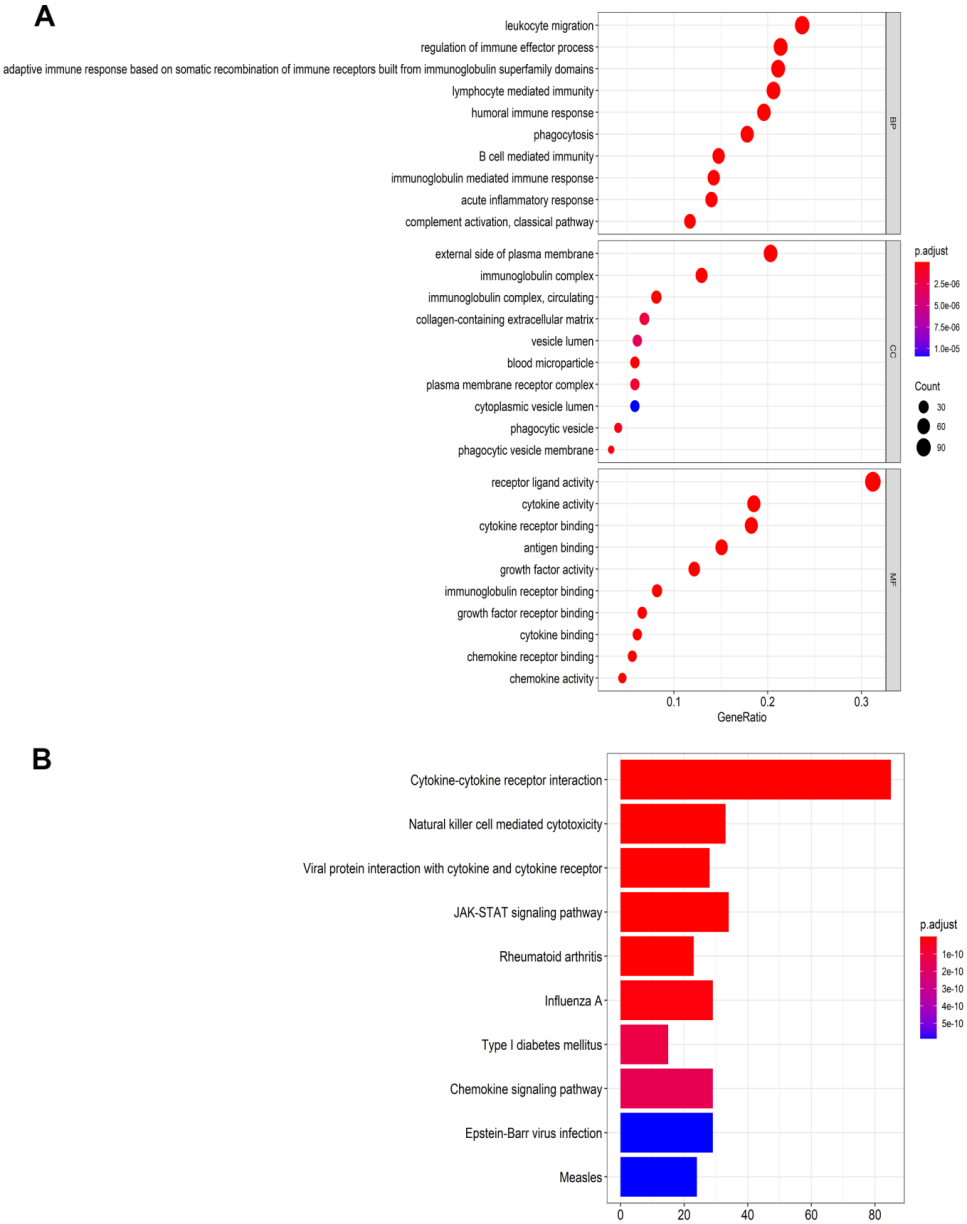
**Functional enrichment analysis of differentially expressed IRGs.** (**A**) The top 10 most significant categories as determined by Gene ontology analysis. The figure represents biological process (BP), cellular component (CC) and molecular function (MF) genes from top to bottom. (**B**) Kyoto Encyclopedia of Genes and Genomes (KEGG) pathways.

For the TCGA dataset, a total of 400 immune-related genes (IRGs) (305 upregulated and 95 downregulated) and 63 transcription factors (TFs) (46 upregulated and 17 downregulated), which were differentially expressed in HNSCC tissues (n=502) compared with adjacent normal tissues (n=44), were identified and presented in heat maps and volcano plots (Supplementary Figure 1). The 400 IRGs were further analyzed by the Gene Ontology (GO) and Kyoto Encyclopedia of Genes and Genomes (KEGG) algorithms and the top 10 results were shown in Figure 1. The GO functional analyses consisted of the following three parts: biological process (BP), molecular function (MF), and cell component (CC). From Figure 1, we identified eight pathways that were enriched in the immune-related gene signature by KEGG analysis. These primarily included genes involved in MAPK signaling, EGFR tyrosine kinase inhibitor resistance, and Ras signaling, and might participate in the development of HNSCC.

### Regulatory network of TFs

**Figure 2:**
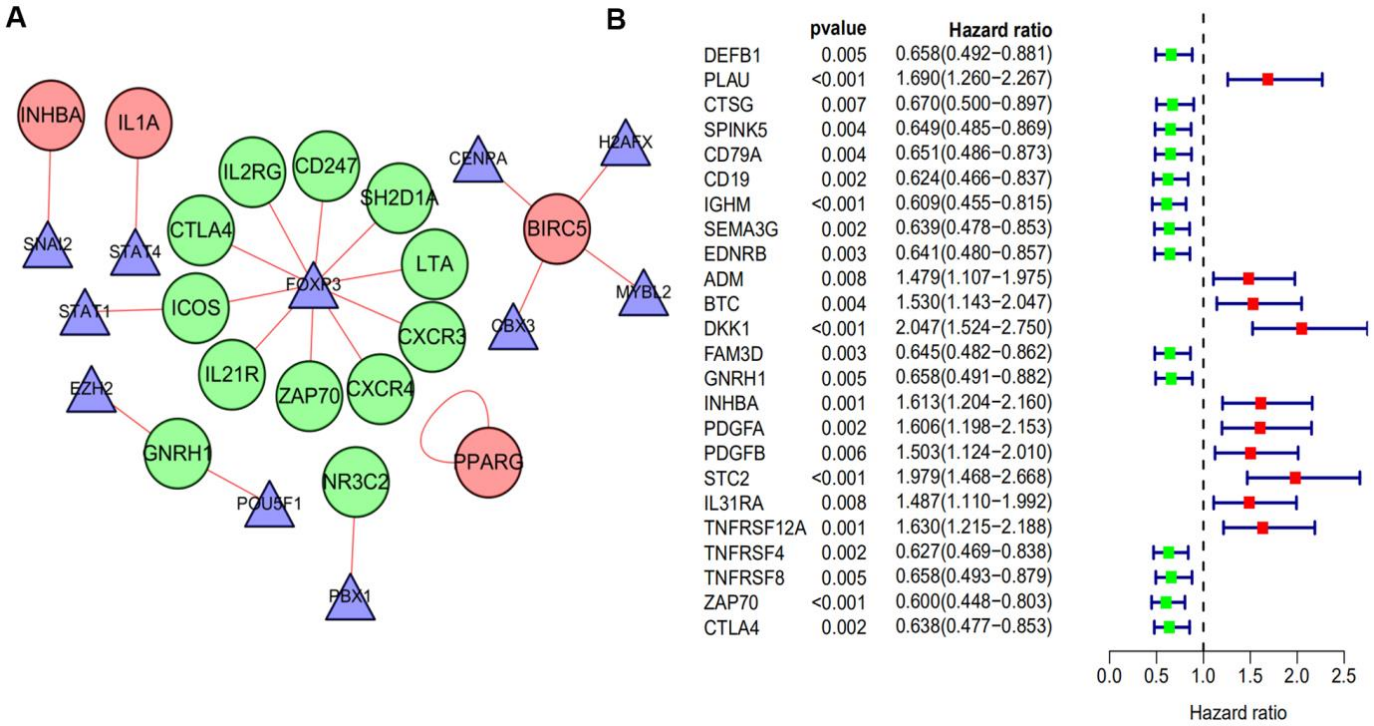
**The regulatory network of TF and univariate cox analysis of differentially expressed IRGs and OS of HNSCC.** (**A**) The regulatory network constructed based on prognosis-related IRGs and TFs. The red circle represents the high-risk IRGs and the green circular represents the low-risk IRGs. The triangle represents differentially expressed TFs. The red link line represents positive regulation, while there was no observed negative regulation. (**B**) A total of 24 differentially expressed IRGs found to be significantly associated with the OS of HNSCC patients (*p*-value < 0.01). Red points represent positive correlations, while green points represent negative correlations.

A total of 51 IRGs that significantly associated with overall survival (OS) were identified by using a log-rank test with a univariate Cox analysis (*p* < 0.05). Then, we identified 51 genes with a *p*-value < 0.05 for analysis with differentially expressed TFs using a Pearson’s correlation test. As a result, correlation coefficients > 0.5 with *p* < 0.05 were used to establish a network, which was done using the Cytoscape software (Figure 2A). As shown in the network, we found that Foxp3 occupied a major position and positively regulated most of the low-risk IRGs including LTA, CXCR4, CXCR3, IL21R, CD247, ZAP70, SH2D1A, ICOS, and CTLA4. This suggests that Foxp3 may play an important role in the immune regulation of HNSCC.

### Construction and validation of the 10 immune-related gene signature

**Figure 3:**
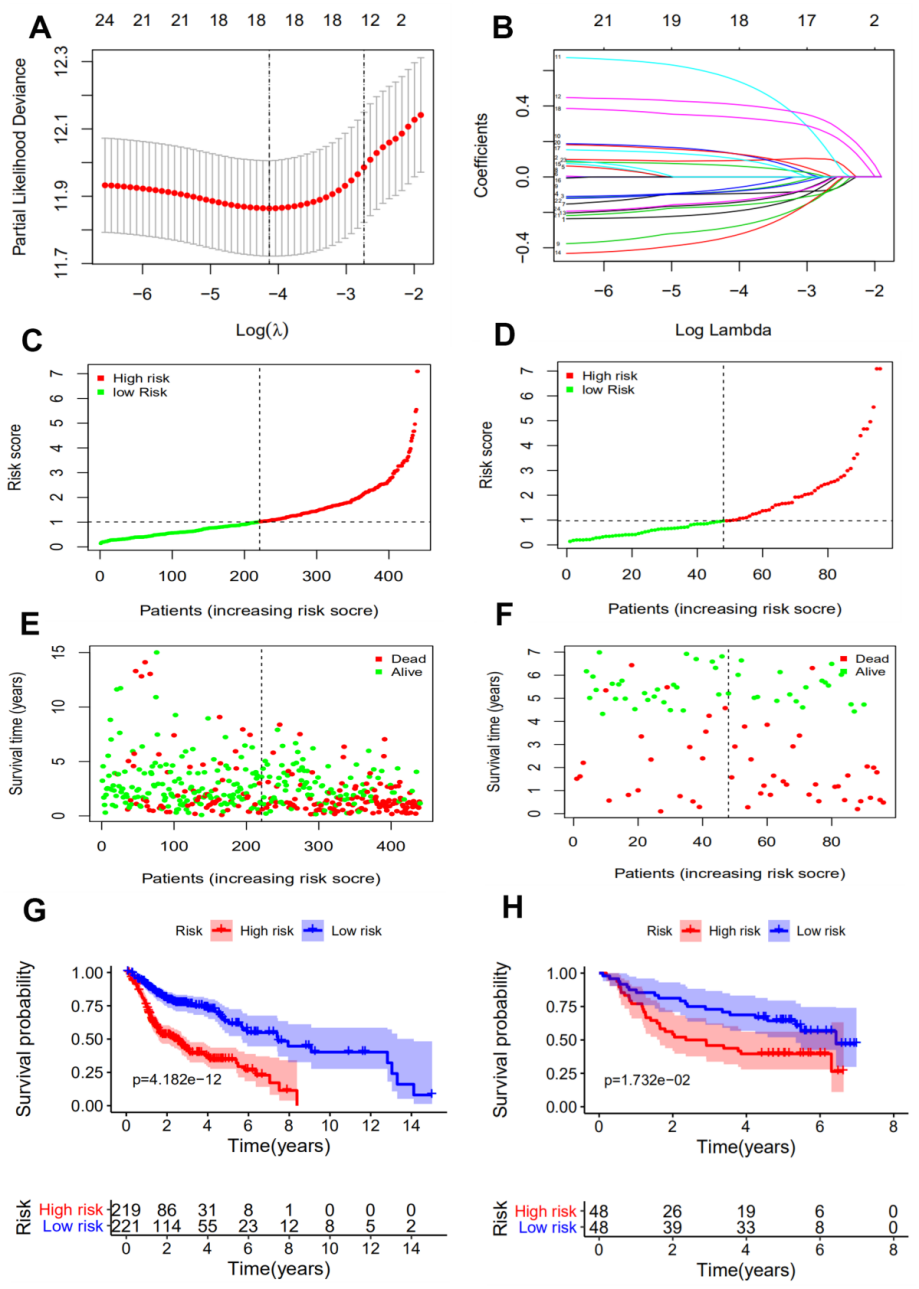
**Construction of an immune-related prognostic signature for HNSCC.** (**A**) Eighteen immune-related genes selected by LASSO Cox analysis. (**B**) A 10-fold cross-validation for the optimal penalty parameter lambda. (**C**, **D**) The risk score distribution of HNSCC patients in the training and validation sets. (**E**, **F**) Survival status and duration of patients in the training and validation sets. (**G**, **H**) Survival curves for the low and high risk groups in the training and validation sets.

The 24 prognostic IRGs, which were obtained from the univariate Cox analysis with *p* value < 0.01 (Figure 2B), were subjected to Lasso analysis and 18 genes were screened out (Figure 3A, 3B). The multivariate Cox regression model was then applied to select the final gene set. As a result, 10 immune-related genes were filtered to establish a prognostic model. The formula for the prognostic model was as follows: risk score = (-0.319322778 * DEFB1 status) + (-0.417843642 * EDNRB status) + (0.24520493 * ADM status) + (0.709588827 * BTC status) + (0.477760262 * DKK1 status) + (-0.25962002 * FAM3D status) + (-0.453322755 * GNRH1 status) + (0.416649823 * STC2 status) + (0.2610282 * TNFRSF12A status) + (-0.357569677 * CTLA4 status). Based on the prognostic model formula, we calculated each patient’s risk score and divided them into high and low risk groups based on the median value of risk scores in the training and validation sets. Then, the risk score and survival status of patients for the 10-gene signature model were determined for the training and validation sets (Figure 3C–3F). Finally, the survival analysis of high and low risk groups in the training and validation sets was presented. Patients in the high risk group had significantly poorer prognosis compared with those in the low risk group (*p* < 0.001; Figure 3G, 3H).

### A 10-gene immune-related signature is an independent prognostic factor

**Figure 4:**
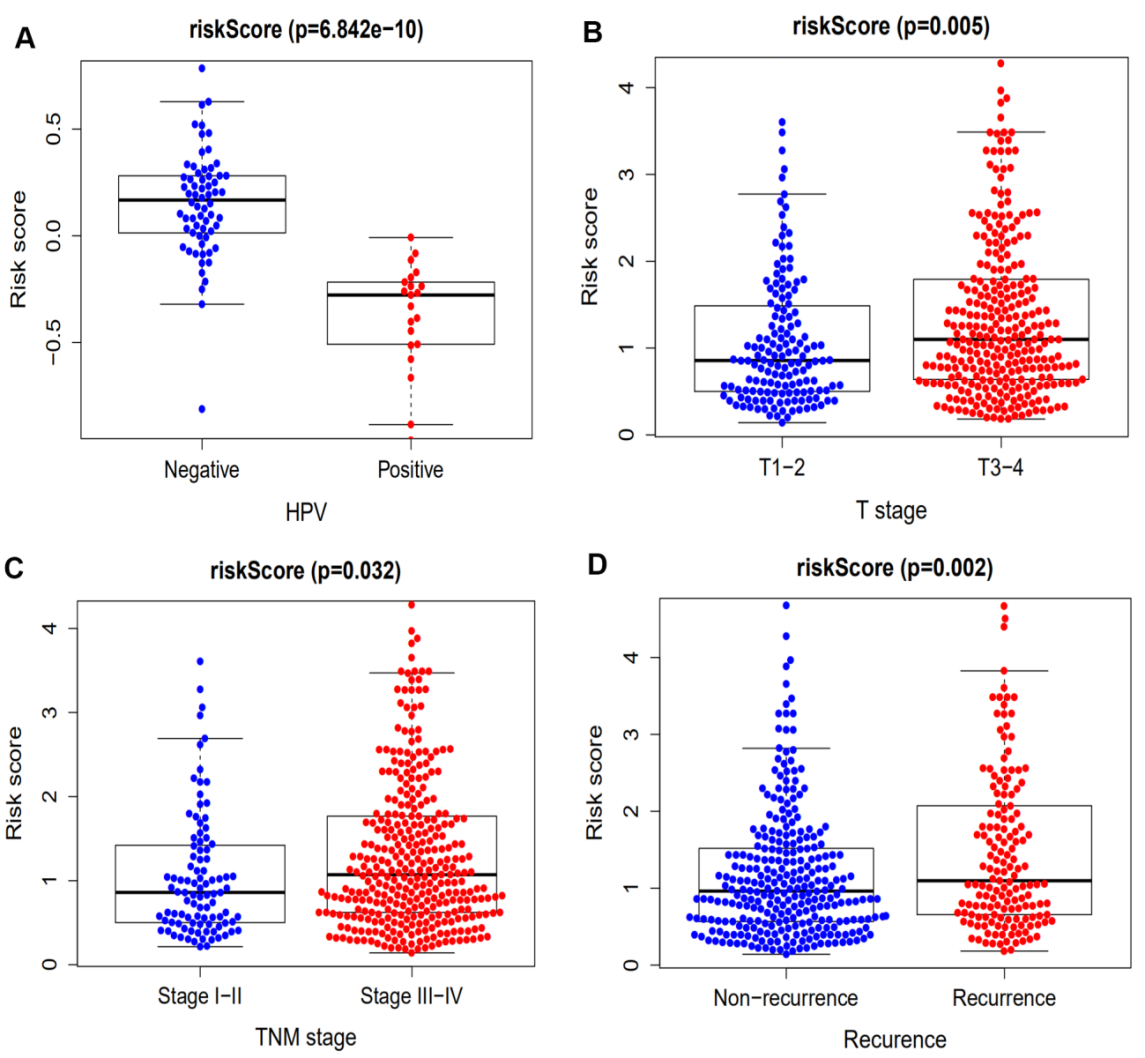
**The association between our prognostic signature and HPV, T stage, TNM stage and recurrence.** (**A**) The HPV-positive patients exhibited a lower risk score compared with HPV-negative patients (*p* = 6.842 x 10^-5^). (**B**) The immune-related signature risk scores for T3 and 4 stage HNSCC patients were notably higher compared with that of T1 and 2 patients (*p* = 0.005). (**C**) The immune-related signature risk scores for stage III & IV HNSCC patients were notably higher compared with that of stage I & II patients (*p* = 0.032). (**D**) Patients with recurrence of HNSCC have higher risk scores compared with those having no recurrence (*p* = 0.002).

To determine whether the 10-gene immune-related signature was an independent prognostic factor for patient survival in the training and validation sets, univariate and multivariate Cox models were established (Table 1). The results of the univariate Cox analysis showed that age, N stage, M stage, recurrence and risk score were the factors associated with patient prognosis in the training set. TNM stage and risk score were the factors associated with patient prognosis in the validation set. In addition, our immune-related signature was an independent prognostic risk factor in both the training set (461 cases: HR=1.495, 95% CI (1.349−1.657), *p* < 0.001) and the validation set (96 cases: HR=1.347, 95% CI (1.107−1.638), *p* = 0.003) by multivariate analysis. The association of our signature with clinicopathologic factors was carried out and the results indicated that only T stage, TNM stage, recurrence, and human papillomavirus (HPV) exhibited statistical significance (*p* < 0.05, Figure 4).

**Table 1:** Univariate and multivariate analyses of overall survival in HNSCC patients in training set and validation set.

**Dataset**	**Characteristics**	**Univariate**		**Multivariate**	
		**HR (95%CI)**	***p*-value**	**HR (95%CI)**	***p*-value**
Training set	Age	1.018(1.004−1.031)	**0.012**	1.021(1.007−1.035)	**0.003**
	Gender	0.811(0.593−1.109)	0.189		
	Grade	1.108(0.891−1.377)	0.357		
	Smoking	0.987(0.870−1.120)	0.841		
	Alcohol	1.106(0.809−1.514)	0.527		
	TNM Stage	1.110(0.942−1.308)	0.211		
	T stage	1.115(0.958−1.298)	0.159		
	N stage	1.201(1.027−1.403)	**0.022**	1.173(1.001−1.374)	**0.049**
	M stage	7.816(2.865−21.320)	**<0.001**	4.672(1.641−13.302)	**0.004**
	Recurrence	3.253(2.430−4.355)	**<0.001**	2.618(1.935−3.543)	**<0.001**
	Risk score	1.603(1.454−1.766)	**<0.001**	1.495(1.349−1.657)	**<0.001**
Validation set	Age	1.005(0.607−1.662)	0.986		
	Gender	1.092(0.602−1.980)	0.771		
	TNM Stage	3.757(1.919−7.357)	**<0.001**	3.469(1.764−6.825)	**<0.001**
	Risk score	1.383(1.152−1.661)	**<0.001**	1.347(1.107−1.638)	**0.003**

*In Cox regression analysis, gender was defined as female=0, male=1; grade, TNM stage and T, N, M stage were defined as 1-4 in accordance with the increase of stage, respectively; smoking, alcohol and recurrence were defined as No=0 and Yes=1, respectively; HR, hazard ratio; CI, confidence interval. Bold values indicate *p* < 0.05, which represents statistical significance.

### External validation of the immune-related gene signature

**Figure 5:**
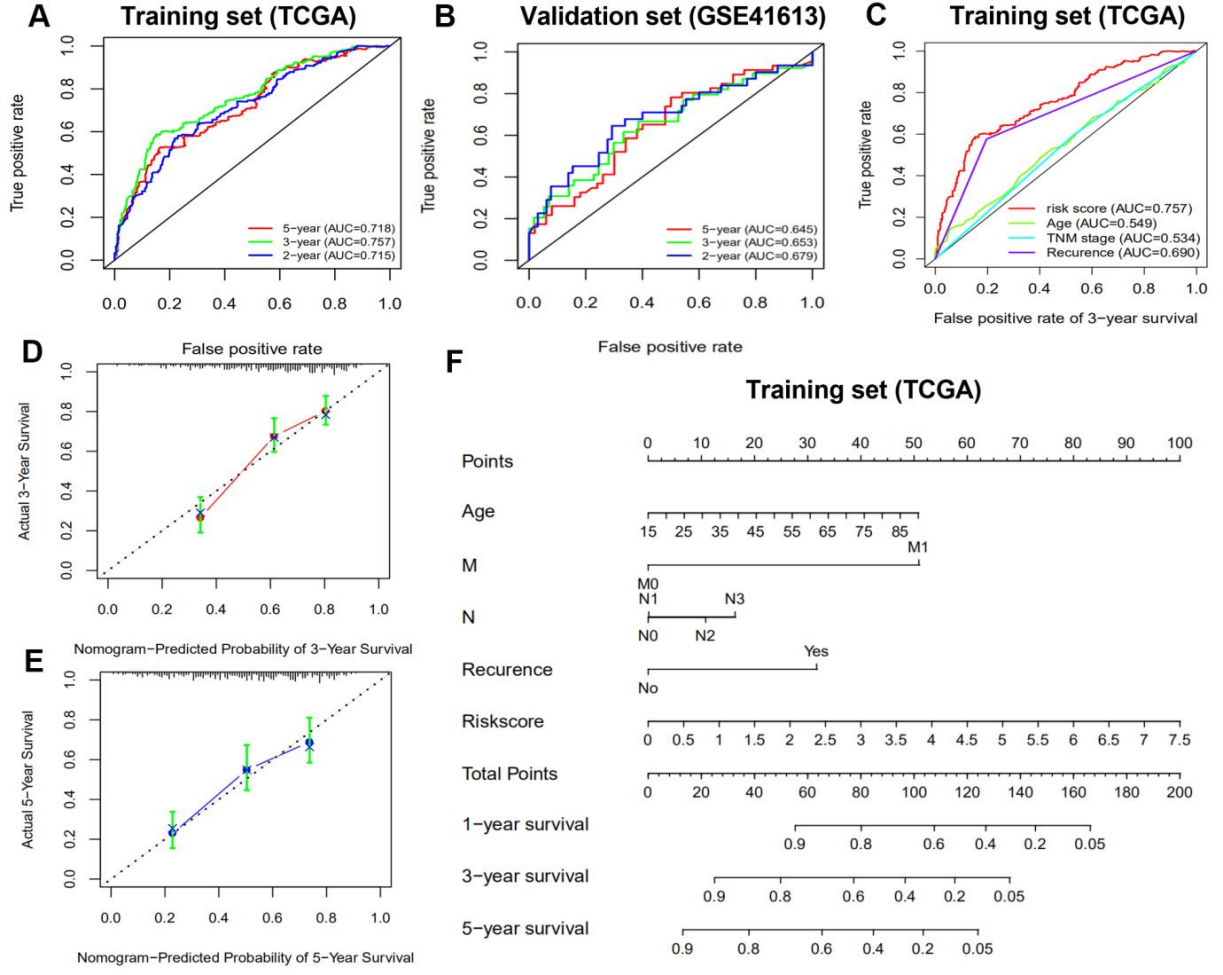
**Receiver operating characteristic curve (ROC) analysis and nomogram construction predicted overall survival using risk score.** (**A**, **B**) ROC analysis for predicting 2-, 3- and 5-year survival times in the training and validation sets. (**C**) ROC analysis between risk score and clinicopathological characteristics in the training set. (**D**, **E**) Calibration curves of the nomogram for predicting the probability of 3- and 5-year survival. (**F**) A nomogram to quantitatively predict 1-, 2-, and 3-year survival for HNSCC patients based on the prognostic signature and clinicopathological characteristics of the training set.

The area under the ROC curve (AUCs) were 0.715, 0.757 and 0.718 for 2-, 3- and 5-year survival times, respectively, for the training set. The AUCs for the validation set were 0.679, 0.653, 0.645 for 2-, 3- and 5-year survival times, respectively, suggesting that our prognostic signature exhibited better sensitivity and specificity (Figure 5A, 5B). Compared with clinicopathologic factors including gender, TNM stage, T stage, N stage, and recurrence, the AUCs of our signature were the highest (Figure 5C). This demonstrated that the 10-gene signature model had a better ability to predict patient prognosis. Furthermore, the nomogram calibration curves for the possibility of 3- and 5-year OS showed consistency between predicted and actual survival in the training set (Figure 5D, 5E). A nomogram with clinicopathologic factors and risk score was established to quantitatively predict the prognosis of HNSCC patients. It revealed that our prognostic signature was a key factor for predicting the survival, while the clinicopathological characteristics showed an inferior impact (Figure 5F).

**Figure 6:**
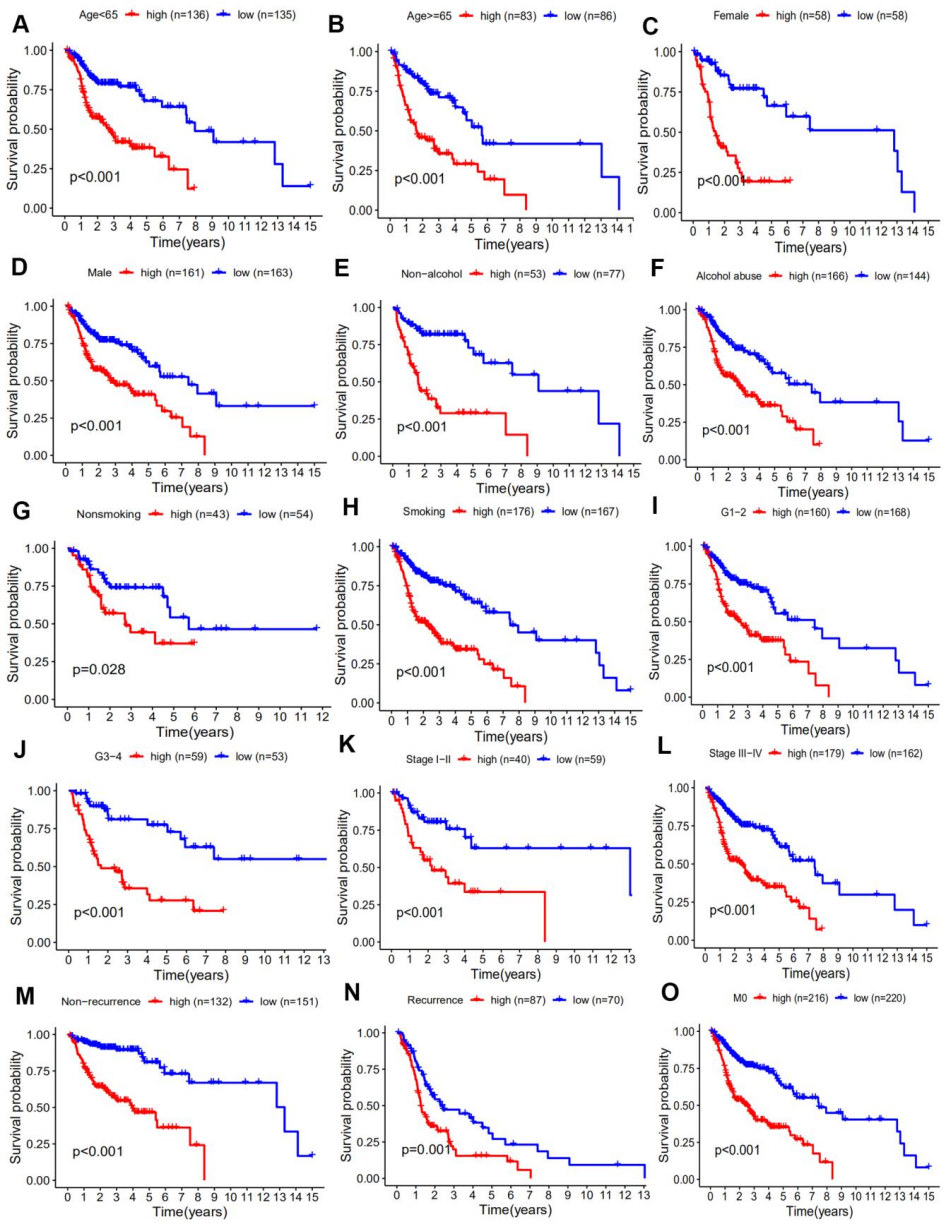
**Kaplan-Meier analyses of HNSCC patient subgroups and clinicopathology factors including.** (**A**) age ≥65 years, (**B**) age < 65 years, (**C**) female, (**D**) male, (**E**) non-alcohol, (**F**) alcohol, (**G**) Nonsmoking, (**H**) smoking, (**I**) G1 and 2, (**J**) G3 and 4, (**K**) stage I-II, (**L**) stage III-IV, (**M**) non-recurrence, (**N**) recurrence, (**O**) M0 stage (*p* < 0.05).

We further determined whether our 10-gene immune-related signature could predict the survival of patient subgroups. We stratified patients on the basis of clinicopathological factors including age, gender, smoking, alcohol, TNM stage, recurrence, and M0 stage. The results revealed that our signature might be an independent and significant prognostic predictor for clinical outcome in patient subgroups (Figure 6). The patients with M1 stage disease were not included because of the small number of cases.

### Association between immune-related gene signature, immune cell infiltration, and immune checkpoint molecules

**Figure 7:**
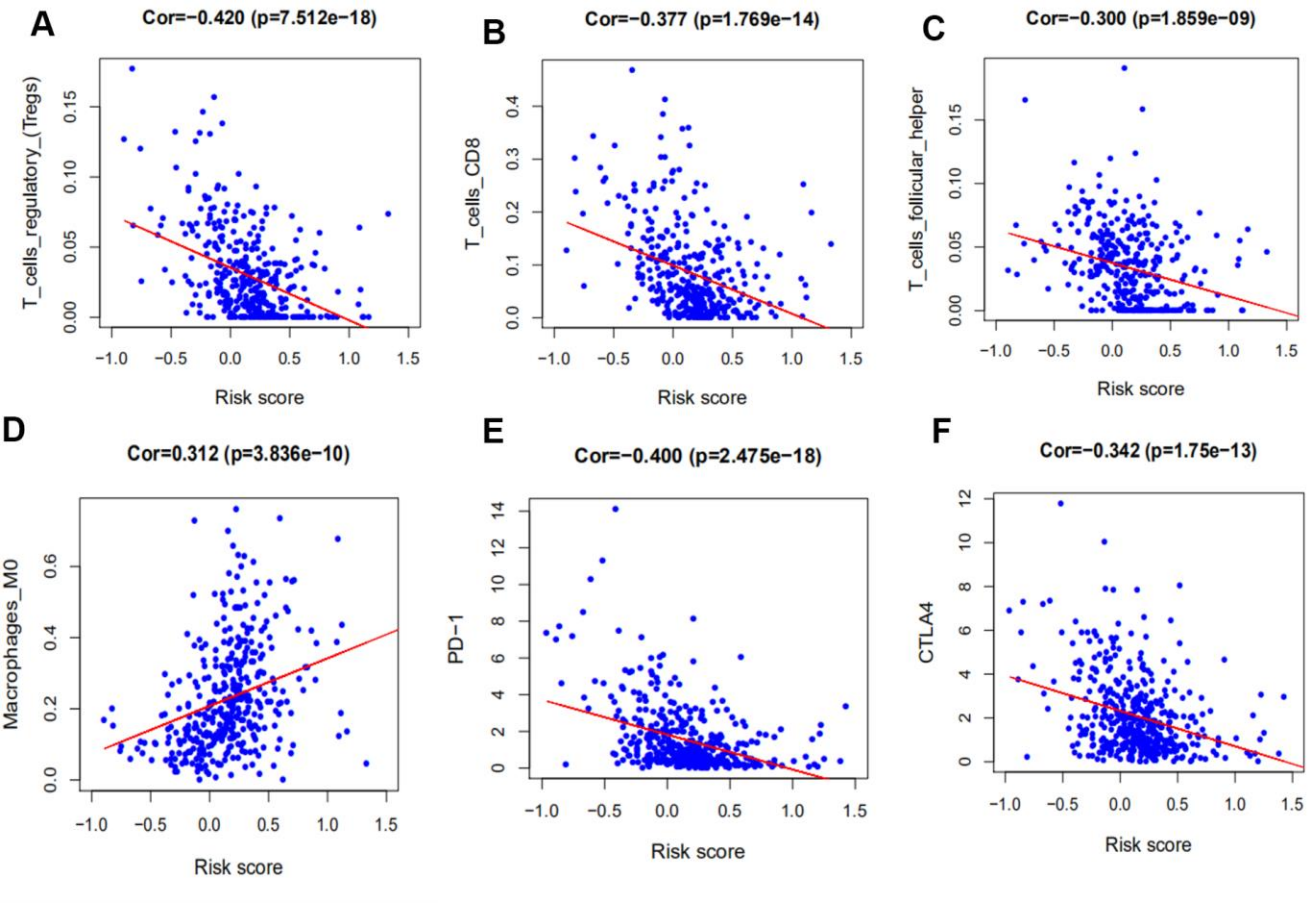
**Correlation between our prognostic signature and that of tumor-infiltrating immune cells and immune checkpoint molecules.** (**A**) Association between risk score and T regulatory cells (Tregs). (**B**) Association between risk score and activated Mast cells. (**C**) Association between risk score and M0 macrophages. (**D**) Association between risk score and CTLA-4. (**E**) Association between risk score and PD-1. (**F**) Association between risk score and PD-L1.

To investigate the association between our immune-related signature and 22 immune infiltration cell types, a correlation analysis was performed with |spearman coefficients| ≥ 0.2 and *p* values < 0.05 (Figure 7A–7C). The relationship between our signature and immune checkpoint molecules was also established (Figure 7D–7F). The results of the Pearson’s correlation analysis indicated that our prognostic signature was negatively associated with regulatory T cells (Tregs) (r = −0.296, *p* < 0.001), while M0 macrophages (r = 0.203, *p* < 0.001) and activated Mast cells (r = 0.204, *p* < 0.001) were positively associated. With an increase in immune risk score, the expression of Tregs decreased gradually in contrast to the signatures of M0 macrophages and activated Mast cells. The prognostic signature was also negatively correlated with immune checkpoint molecules, including CTLA-4 (r = 0.253, *p* < 0.001) and PD-1 (r = 0.198, *p* < 0.001), but not with PD-L1 (*p* > 0.05).

### Genetic alterations and GSEA analysis in the high-risk groups

**Figure 8:**
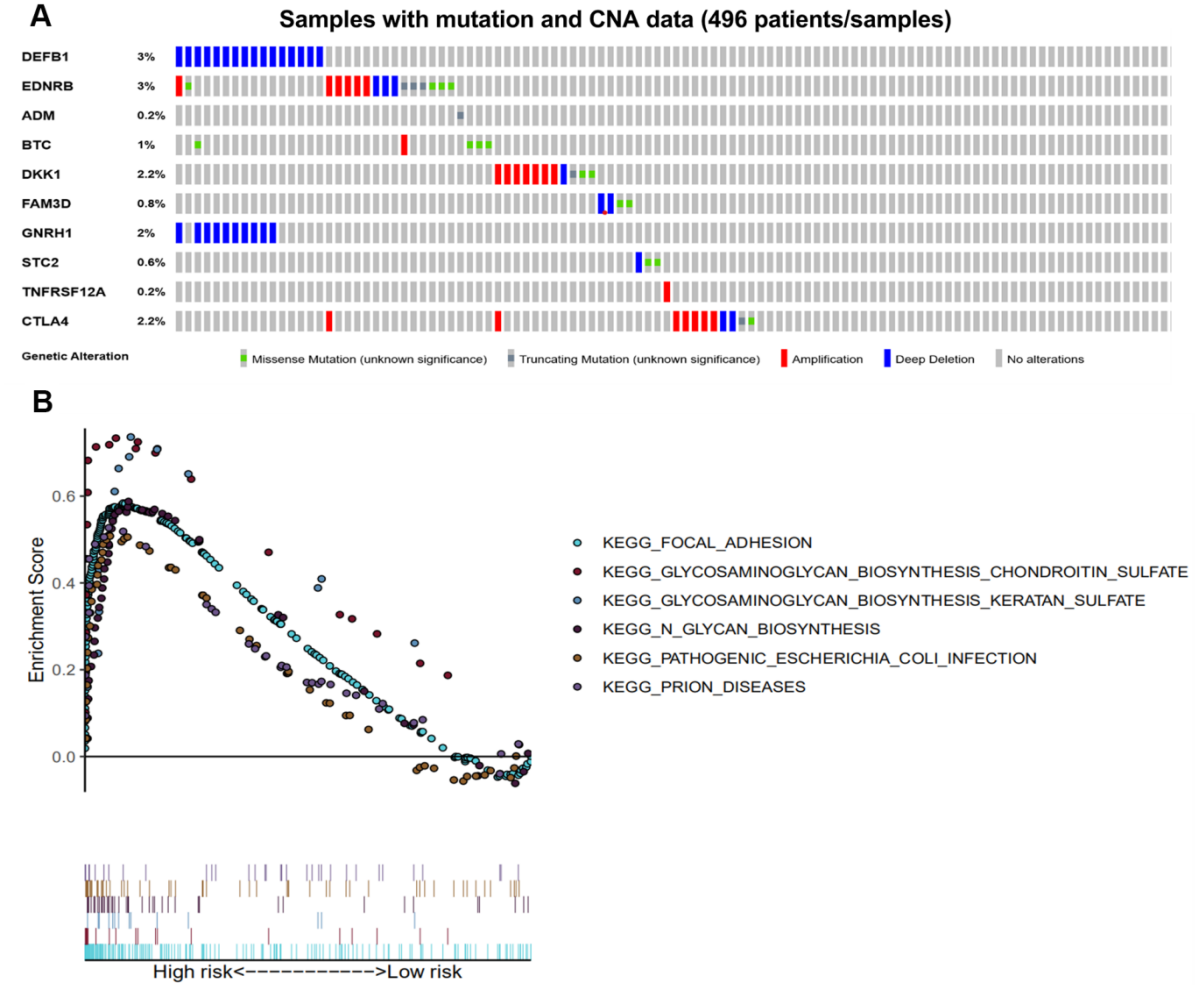
**Genetic alterations of the 10 immune-related genes using GSEA.** (**A**) Genetic alterations of 10 immune-related genes in HNSCC samples. The rows and columns indicate the genes and tumor samples, respectively. (**B**) The six enriched pathways in the high-risk groups based on the prognostic signature in HNSCC.

The genomic alterations of our 10 immune-related genes in each patient were analyzed using the cBioPortal tool (Figure 8A). The genetic alteration percentages ranged from 0.2-3%, and likely have little influence on mRNA expression. The 10 immune-related genes were altered in 62 (13%) of the 496 patients. DEFB1 and GNRH1 were primarily affected by deep deletion, while the CTLA-4, DKK1 and EDNRB were frequently amplified. The pathways enriched in the high-risk group of the training set were analyzed by GSEA. As a result, there were six pathways that were significantly enriched in the high-risk patients (Table 2 and Figure 8B).

**Table 2:** The six enriched pathways in the low-risk group.

**NAME**	**SIZE**	**ES**	**NES**	**NOM *p*-val**	**FDR q-val**	**FWER *p*-val**
KEGG_GLYCOSAMINOGLYCAN_BIOSYNTHESIS_KERATAN_SULFATE	15	0.737	1.941	0.000	0.071	0.085
KEGG_PRION_DISEASES	35	0.527	1.753	0.004	0.136	0.349
KEGG_PATHOGENIC_ESCHERICHIA_COLI_INFECTION	56	0.508	1.835	0.004	0.091	0.204
KEGG_FOCAL_ADHESION	199	0.584	1.956	0.004	0.116	0.070
KEGG_GLYCOSAMINOGLYCAN_BIOSYNTHESIS_CHONDROITIN_SULFATE	22	0.734	1.927	0.006	0.052	0.090
KEGG_N_GLYCAN_BIOSYNTHESIS	46	0.588	1.846	0.008	0.104	0.193

## DISCUSSION

Head and neck squamous cell carcinoma is considered to be a heterogeneous disease and its biological behavior is frequently aggressive. The high mortality rate observed for HNSCC is primarily due to the frequent recurrence of advanced tumors [17]. There is a significant need for clinicians to give personalized and realistic prognostic prediction as TNM stage is no longer an accurate prognostic indicator. Therefore, it is crucial to identify new markers that predict clinical outcome, achieve personalized approaches to therapy, and establish early intervention treatments. To date, many studies have tried to establish prognostic signatures, including gene sets [18], miRNAs [19], lncRNAs [20] and methylation analyses [21], as promising predictors of prognosis for HNSCC. In recent years, the immune system has been recognized as playing an important role in cancer development and progression [22]. Nevertheless, the contribution of immune-related molecular mechanisms to HNSCC remain unclear.

In the present study, we screened 400 differentially expressed immune-related genes from the TCGA dataset. Using GO and KEGG enrichment analysis, we found that the immune-related genes were primarily associated with immune response, cancer, and drug resistance pathways (i.e. MAPK signaling pathway, EGFR tyrosine kinase inhibitor resistance, Ras signaling pathway and endocrine resistance). Similar studies have demonstrated that the MAPK signaling pathway participates in cancer progression, including proliferation, apoptosis and immune escape, and it is fundamental to cancer control [23].

Transcription factors regulate gene expression and their dysregulation or mutation is well known to contribute to tumorigenesis [24]. Foxp3, a member of the forkhead transcription factor family, is one of the key transcription factors that controls the development and function of Treg cells [25]. An analysis of the TF-mediated network was done to reveal the regulatory mechanisms of prognostic immune-related genes. The results indicated that Foxp3 was a crucial TF that upregulated most of the low-risk prognostic immune-related genes. This suggested that Foxp3 might be a key factor in the immune regulatory mechanism of HNSCC. Foxp3 might also control the immune microenvironment by regulating the expression of genes that contribute to the immunotherapy of HNSCC. Foxp3 and CTLA4 were also determined to be positively correlated in our study, consistent with that of previous studies [26, 27].

It has been demonstrated that immune gene signatures can predict prognosis in many solid tumors including ovarian cancer [28], clear cell renal cell carcinoma [29], cervical cancer [30], lung adenocarcinoma [31], and hepatocellular carcinoma [32]. In the present study, we developed a prognostic signature based on 10 immune-related genes from TCGA dataset and validated them using GSE41613 dataset. The patients in the high risk group were considered to have short survival times in both datasets, in accordance with previous studies [11]. ROC analysis indicated that our immune signature exhibited better sensitivity and specificity for survival prediction at 2-, 3- and 5-years, even exceeding the predictive ability of TNM stage. Moreover, multivariate Cox analysis indicated that the 10-gene immune-related signature was an independent prognosis risk factor for HNSCC. Patients exhibiting recurrence had higher risk scores compared with patients without recurrence, suggesting that the prognostic signature had a broader predictive value for recurrence. A previous study demonstrated that HPV-positive HNSCC patients had improved survival compared with HPV-negative patients [33]. This is consistent with our findings that HPV-positive patients had a lower risk score. We further predicted survival time of patients with different clinical factors based on our 10 immune-related gene signature. This demonstrated that our signature could act as accurately and strongly biomarkers for predicting prognosis in HNSCC patients with various clinicopathologic factors.

Other studies corroborate that our 10 immune-related gene signature is closely related to the development of cancer. For example, DEFB1 (encoding human ß-defensin-1 [HBD-1]), is a potential tumor suppressor which has been shown to participate in the innate immune response and can suppress tumor migration and invasion in oral squamous cell carcinoma [34]. EDNRB promoter methylation, which is associated with the histologic diagnosis of premalignancy and the presence of malignancy, may be a promising marker for the early detection of premalignant lesions in oral cavity cancer [35]. The ADM gene, which plays a role in carcinogenesis by regulating cellular processes including proliferation, differentiation, migration, growth, immunosuppression and hypoxia, increases lymph node metastasis risk in oral and oropharynx cancer [36]. Increased BTC mRNA expression is associated with worse survival in HNSCC [37]. Dkk-1, a tumor suppressor gene, is associated with distant metastasis in HNSCC patients, when happens to allelic loss at Dkk-1 locus frequently [38]. Overexpression of FAM3D-AS1 is demonstrated to inhibit cell proliferation, invasion, EMT, and cell survival rate in colorectal cancer [39]. The level expression of GNRH1 has been shown to indicating metastatic spread of tumor cells in gynecological malignances [40]. The downregulation of STC2 plays a vital role in the metastasis and progression of HNSCC [41]. TNFRSF12A contributes to carcinogenesis by promoting angiogenesis, proliferation, apoptosis, migration and inflammation in tumors, can cause cachexia, and is a promising therapeutic target to prolong survival [42]. CTLA-4, a checkpoint for tumor immunotherapy, can induce T cells to be nonreactive and participate in the repression of T cell proliferation, cell cycle progression, and the immune response [43].

To further understand the relationship of immune-related prognostic signature and immune microenvironment, 22 immune infiltrating cells, derived from CIBERSORT, and 3 immune checkpoint molecules were selected for analysis. In cancer, Tregs contribute to tumor immune escape by suppressing the antitumor response. Some studies had reported that higher Foxp3+ Treg cell infiltration was associated with poorer patient survival in most tumors, but not in HNSCC [44, 45]. Our results showed that the high level of Tregs infiltration was significantly associated with the low risk score, which was associated with favorable prognosis. Macrophages that infiltrate into the tumor microenvironment may facilitate tumor growth, angiogenesis, invasion, and metastasis, and are associated with poor prognosis in HNSCC [46]. Our study indicated that they were positively associated with risk score and an increase in M0 macrophages portended a poor prognosis.

Recently, the immunocheckpoints involving PD-1, PD-L1, and CTLA-4 represent promising immunotherapy targets for antitumor therapy [47]. PD-1 is a transmembrane protein that is mainly expressed on the surface of T lymphocytes. CTLA-4 is a membrane glycoprotein that is frequently expressed on Tregs. The mechanism of action of CTLA-4 and PD-1 remain controversial. Our study revealed that high CLTA-4 expression was significantly associated with a lower risk score. This may be caused by the upregulation of Tregs in HNSCC, suggesting that CTLA-4 may be involved in some aspect of the antitumor effect. Studies have reported that HNSCC patients exhibiting high PD-L1/PD-1 expression tend to have prolonged survival outcomes and a lower probability of recurrence [48, 49]. In addition, our signature was negatively correlated with PD-1, but not PD-L1.

Studies have found that the genetic variation of HBD-1 contributes to lower RNA expression and may be involved in carcinogenesis of oral squamous cell carcinoma [50]. The genetic alterations of our 10 immune-related genes may help explain the aberrant expression of these genes to some extent in tumors, and patients that carry such genetic alterations may be more responsive to immunotherapy. Meanwhile, our GSEA results indicated that six enriched pathways in the high-risk immune group were significantly correlated with the biological processes in HNSCC progression. For example, the pathway of focal adhesion in HNSCC participates in the development of distant metastasis to lymph nodes [51]. The keratan sulphate in the tumor environment plays a vital role in the promotion or regulation of tumor development [52]. The chondroitin sulfate glycosaminoglycan biosynthesis pathway can promote and regulate tumor progression and metastasis by influencing important biological processes such as cell growth, adhesion, signal transduction, and lipid metabolism [53].

The above results demonstrate that our signature has potential clinical prognostic value and is associated with response regulated by the immune microenvironment. This may provide a potential target for diagnosis and for development of new targeted therapies.

There are several limitations to our study. Firstly, our study was performed using public databases and had a retrospective design, so further studies should be done with prospective clinical data sets to validate our results. Secondly, although we established and verified our model using different gene expression datasets, concerns regarding sample bias and model over-fitting still remained. Furthermore, the infiltrating cell populations were calculated by an analytical tool (CIBERSORT) using gene expression data. This is different from the patient's tumor cell infiltration and thus have the false discovery rate. Finally, the biological functions of our immune signature should be further validated in biological experiments.

## MATERIALS AND METHODS

### Study samples

The training set was acquired from the TCGA data portal (https://portal.gdc.cancer.gov/cart; up to March 22, 2020), and consisted of processed RNA-Seq FPKM data for HNSCC patients (n=461). The corresponding clinical data, including age, gender, smoking, alcohol abuse, differentiation grade, clinical TNM stage, T stage, N stage, M stage, recurrence, and survival information were completely provided. Next, data from 96 cases (GSE41613) were downloaded from the GEO database (https://www.ncbi.nlm.nih.gov/geo/) along with clinical data and follow-up time as a validation set. Only patients with complete clinical and expression data available at that time were included in this study and the survival time threshold in our study was greater than one month. The clinicopathological characteristics of HNSCC patients from the training and validation sets were showed (Supplementary Table 1). The OS was defined as the date of the study enrollment to the last follow-up time.

### Differentially expressed genes of IRGs and TFs

We obtained IRGs from the Immunology Database and Analysis Portal (ImmPort) (https://www.immport.org/) and TFs data were downloaded from the Cistrome Cancer resource (http://cistrome.org/CistromeCancer/CancerTarget/). To establish a training set, we used the R package Limma to identify differentially expressed genes (DEGs) for IRGs and TFs from 502 HNSCC tissues and 44 adjacent normal tissues in TCGA dataset, where FDR < 0.05 and |log(FC)| ≥ 1 were set as the screening criteria [54]. Heat maps and volcano plots of IRGs and TFs were also generated with R software. Furthermore, we assessed the biologic functions of differentially expressed IRGs using the GO and KEGG pathway databases. Enrichment analysis was done with the Cluster Profiler package [55] in R and functional categories with *p* < 0.05 were shown.

### Regulatory TF networks

To further investigate the relationship between DEGs of IRGs and HNSCC prognosis, we used the univariate Cox proportional hazard model. Genes with a *p*-value < 0.05 were selected for further analysis and genes with a hazard ratio (HR) value > 1 were defined as high-risk IRGs, whereas the remainder were considered low-risk. Finally, the association between the above prognosis IRGs and differentially expressed TFs was analyzed by Pearson’s correlation test. The cut-off criteria included correlation coefficients > 0.5 and *p*-values < 0.05, which were determined by the cor.test function in R (Supplementary Table 2). To more clearly express the relationships, we used Cytoscape for constructing and visualizing the regulatory network [56].

### Construction and validation of an immune-related signature

First, to normalize the differentially expressed IRGs values in the training and validation sets, gene expression values lower than the median were defined as 0, otherwise a value of 1 was assigned. Second, to construct an immune-related prognostic signature, we selected the differentially expressed IRGs with a *p*-value < 0.01 by univariate cox analysis. We then used LASSO Cox regression and multivariate Cox regression model to assess the relationship between prognostic immune-related gene expression and OS in the training set using the survival and glmnet R packages. The smallest parameter model for the immune-related prognostic signature was constructed with a 10-fold cross-validation and used one standard error of the best penalty parameter λ to prevent overfitting [57]. Finally, the risk score for each patient was calculated by gene expression and its corresponding coefficients from the multivariate Cox regression analysis. Patients were then divided into high and low risk groups based on the median risk score. To validate the immune-related prognostic signature, we used the same formula as the training set to calculate each patient risk score followed by classification into high and low risk groups.

A prognostic nomogram combined with prognostic clinicopathological factors, including age, N stage, M stage, recurrence, and immune-related gene signature was constructed using the rms R package. To further validate the prognostic value of our signature, Kaplan-Meier analysis of OS in HNSCC patients with subgroup clinicopathological factors was performed.

### Correlation analysis of infiltrating immune cells and immune checkpoint genes

CIBERSORT is an analytical tool that can provide an estimation of the abundances of member cell types in a mixed cell population by using gene expression data. In this study, we identified 22 immune infiltrating cell types by uploading the gene expression training set to the CIBERSORT webpage (https://cibersort.stanford.edu/) and using a reference LM22 expression signature with 100 permutations. The infiltrating immune cells derived from CIBERSORT included T cells (CD4+ T cells, CD8+ T cells, naïve CD4+ T cells, resting memory CD4+ T cells, γδ T cells, regulatory T cells, follicular helper T cells and regulatory T cells), B cells (naïve B cells, memory B cells and plasma cells), myeloid subsets (M0 macrophages, M1 macrophages, M2 macrophages, activated and resting dendritic cells, activated and resting mast cells, monocytes, neutrophils and eosinophils), and NK cells (activated and resting NK cells). In addition, only the results of the infiltrating immune cell fractions with a *p*-value < 0.05 were considered for further analysis. Finally, the correlations between the immune-related signature and tumor-infiltrating immune cells, immune checkpoint modulators, such as PD-1, PD-L1 and CTLA-4, were determined.

### Mutation characteristics and gene set enrichment analysis of the immune-related signature

The mutation characteristics of our immune-related signature in all HNSCC patients from the TCGA dataset were obtained using cBioPortal (http://www.cbioportal.org/). GSEA was performed to identify the pathways that were significantly enriched between high risk groups based in the immune-related signature. An FDR < 0.25 and nominal *p* < 0.05 were used as the screening criteria to identify significant gene sets by the GESA software.

### Statistical analysis

Kaplan–Meier analysis was done to compare the OS between high and low-risk groups using the log-rank test. Meanwhile, ROC curve was used to evaluate the accuracy of the immune-related gene signature. The clinicopathological characteristics on OS was determined by univariate and multivariate analyses on the basis of the Cox proportional hazards model for both training and validation sets. Furthermore, the correlation between immune-related signature and tumor-infiltrating immune cells and immune checkpoint molecules was evaluated by Pearson’s correlation test. All analyses were conducted using R software (version 3.6.2) and the results were considered significant when corresponding *p*-values < 0.05.

## Supplementary Material

Supplementary Figure 1

Supplementary Tables
