# Supplementary material for: A novel comprehensive immune-related gene signature as a promising survival predictor for the patients with head and neck squamous cell carcinoma
Source: Aging (Albany NY). 2021 Apr 17;13(8):11507–27. doi: 10.18632/aging.202842 (PMC8109104; doi:10.18632/aging.202842)
Supplement: Supplementary Figure 1 [file aging-13-202842-s001.pdf]

SUPPLEMENTARY FIGURE

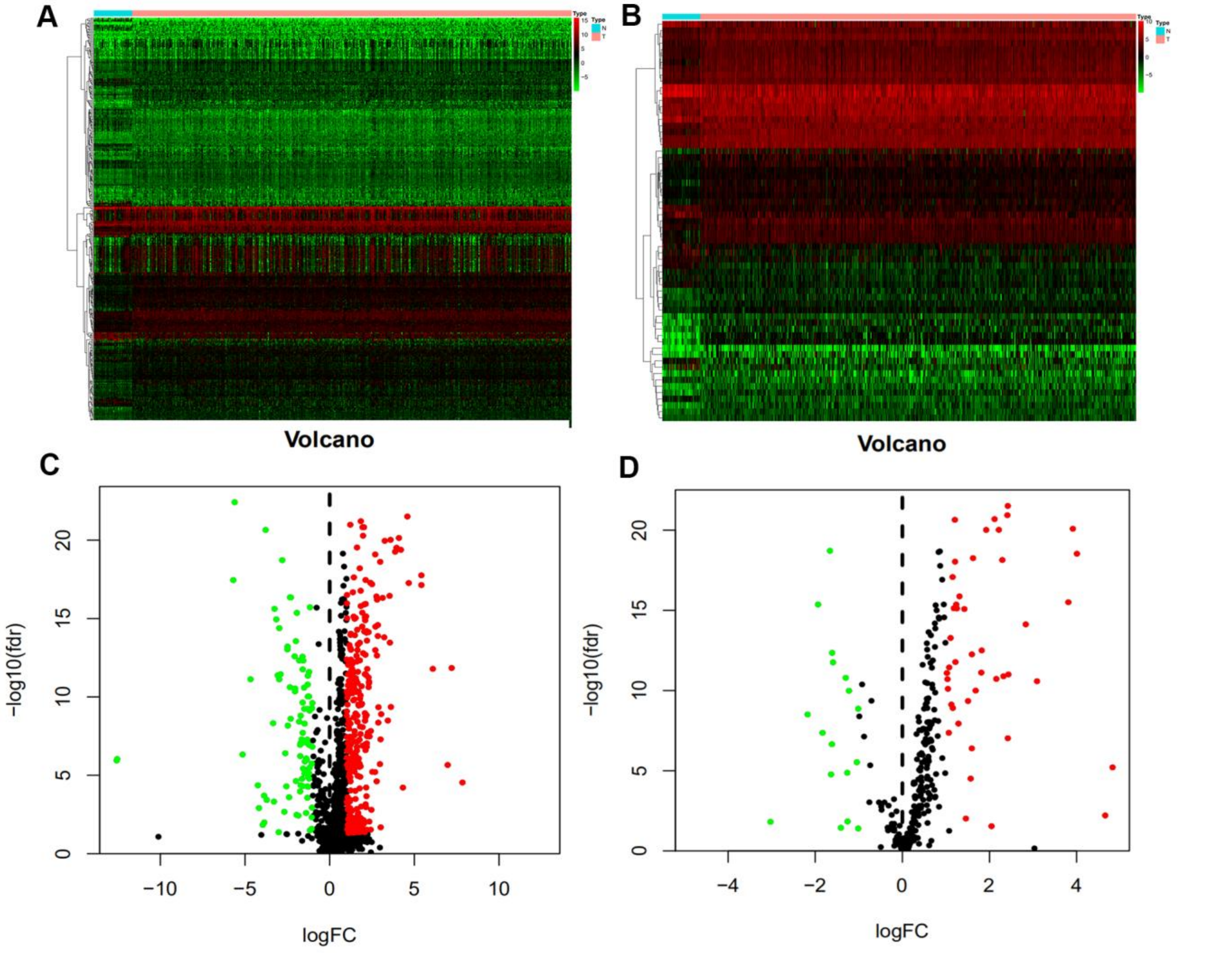

Supplementary Figure 1. Differentially expressed IRGs and TFs in HNSCC. (**A**, **B**) Heat map of significantly differentially IR and TF genes in HNSCC. The color from green to red refers to the progression from low to high expressed genes. (**C**, **D**) Volcano plot of differentially expressed IRGs and TFs. The red and green dots in the plot represent significantly upregulated and downregulated genes, respectively, and no differential expression is depicted by black dots.
