# Supplementary material for: A novel comprehensive immune-related gene signature as a promising survival predictor for the patients with head and neck squamous cell carcinoma
Source: Aging (Albany NY). 2021 Apr 17;13(8):11507–27. doi: 10.18632/aging.202842 (PMC8109104; doi:10.18632/aging.202842)
Supplement: Supplementary Tables [file aging-13-202842-s002.pdf]

SUPPLEMENTARY TABLES

Supplementary Table 1. The clinicopathological characteristics of HNSCC patients from the training and validation sets.

Clinicopathologic parameters	Training set (n = 461) number of cases (%)	Validation set (n = 96) number of cases (%)
**Gender**		
Male	339 (26.46)	65 (67.71)
Female	122 (73.54)	31 (32.29)
**Age**		
<60 y	202 (43.82)	50 (52.08)
≥60 y	259 (56.18)	46 (47.92)
**TNM stage**		
I-II	104 (22.56)	41 (42.71)
III-IV	357 (77.44)	55 (57.29)
**Drinking**		
Yes	328 (71.11)	
No	133 (28.89)	
**Smoking**		
Yes	106 (23.00)	
No	355 (77.00)	
**Grade**		
1-2	340 (73.75)	
3-4	121 (26.25)	
**Lymph node metastasis**		
N0	226 (50.98)	
N+	235 (49.02)	
**T classification**		
T1-T2	161 (34.92)	
T3-T4	300 (65.08)	
**M classification**		
M0	456 (98.92)	
M1	5 (1.08)	
**Recurrence**		
Yes	162 (35.14)	
No	299 (64.86)	

Supplementary Table 2. The association between the prognosis immune-related genes and differentially expressed TFs.

TF	ImmuneGene	Correlation coefficients	P-value	Regulation
CBX3	BIRC5	0.518642408820594	1.15520246900326e-31	positive
CENPA	BIRC5	0.69287091986189	3.39740962361839e-64	positive
EZH2	GNRH1	0.523031102481409	2.89994062578473e-32	positive
FOXP3	LTA	0.735231898437069	5.50266657984769e-76	positive
FOXP3	CXCR4	0.602664676810251	7.50120067401527e-45	positive
FOXP3	CXCR3	0.734367694212062	1.00840545610555e-75	positive
FOXP3	IL21R	0.797986384807953	2.2645404903834e-98	positive
FOXP3	IL2RG	0.664185745269767	2.60811386532034e-57	positive
FOXP3	CD247	0.55309782116114	1.27354006034913e-36	positive
FOXP3	ZAP70	0.532205241805523	1.50989089544079e-33	positive
FOXP3	SH2D1A	0.683751210147642	6.39922931062554e-62	positive
FOXP3	ICOS	0.859952634007701	5.02642593010807e-130	positive
FOXP3	CTLA4	0.733274069140433	2.16300325109839e-75	positive
H2AFX	BIRC5	0.534652159139509	6.7600337274877e-34	positive
MYBL2	BIRC5	0.563377513450786	3.24548208832276e-38	positive
PBX1	NR3C2	0.601407493116652	1.26429217741422e-44	positive
POU5F1	GNRH1	0.609650592416114	3.95154070696453e-46	positive
